# The Evaluation of SHAPE-MaP RNA Structure Probing Protocols Reveals a Novel Role of Mn^2+^ in the Detection of 2′-OH Adducts

**DOI:** 10.3390/ijms24097890

**Published:** 2023-04-26

**Authors:** Kamilla Grzywacz, Agnieszka Chełkowska-Pauszek, Marianna Plucinska-Jankowska, Marek Żywicki

**Affiliations:** 1 Institute of Bioorganic Chemistry Polish Academy of Sciences, 61-704 Poznań, Poland; 2 Department of Computational Biology, Institute of Molecular Biology and Biotechnology, Adam Mickiewicz University, 61-614 Poznań, Poland

**Keywords:** RNA structure probing, SHAPE-MaP, 1M7, BzCN, mutational profiling

## Abstract

Chemical probing, for decades, has been one of the most popular tools for studying the secondary structure of RNA molecules. Recently, protocols for simultaneous analysis of multiple RNAs have been developed, enabling in vivo transcriptome-wide interrogation of the RNA structure dynamics. One of the most popular methods is the selective 2′-hydroxyl acylation analyzed by primer extension and mutational profiling (SHAPE-MaP). In this study, we describe the evaluation of this protocol by addressing the influence of the reverse transcription enzymes, buffer conditions, and chemical probes on the properties of the cDNA library and the quality of mutational profiling-derived structural signals. Our results reveal a SuperScript IV (SSIV) reverse transcriptase as a more efficient enzyme for mutational profiling of SHAPE adducts and shed new light on the role of Mn^2+^ cations in the modulation of SSIV readthrough efficiency.

## 1. Introduction

The RNA structure is the primary feature determining the RNA function. The functional structural motifs within RNA molecules allow for specific interactions with other cellular components, such as proteins, RNAs, or small metabolites [1]. It also determines the faith of the transcripts, providing recognition signals for the processing or the degradation machinery [2,3]. Thus, solving the RNA structure adds ultimate knowledge about RNA biology.

The most informative for the RNA function is its tertiary structure. It illustrates the final arrangement of the RNA chain in space, demonstrating which nucleotides are available for interactions and which are accountable for intra-molecular contacts contributing to RNA stability. Solving the tertiary RNA structure is much more complex than proteins, mostly due to RNA molecules’ higher flexibility and dynamics. Thus, the secondary structure is often used to describe the functional motifs in RNA research. It is determined by a pattern of base pairs observed within the RNA molecule. 

There are several methods available for the determination of the RNA secondary structure. Some of the most widely used methods are based on the mapping of the structurally accessible RNA regions, using chemical reagents which induce cleavage at single-stranded RNA regions (hydroxyl radicals, lead ions) or introduce chemical modifications which can be detected by primer extension (dimethyl sulfate, CMCT, kethoxal) [4]. Furthermore, SHAPE (selective 2′-hydroxyl acylation analyzed by primer extension) has emerged as a valuable strategy for probing the RNA structure. SHAPE uses small hydroxyl-selective electrophilic reagents to probe the reactivity of the RNA ribose 2′-OH group [5,6]. SHAPE chemistry provides quantitative, reproducible, single-nucleotide resolution data because almost all ribonucleotides possess a free 2′-hydroxyl, and each position in the RNA is interrogated. The initial electrophile developed for SHAPE, N-methylisatoic anhydride (NMIA), is not very reactive; therefore, the RNA structures must be probed over tens of minutes. Due to the dynamic nature of the RNA structure, this issue represented a major disadvantage for the initial version of SHAPE chemistry. However, several superior reagents for SHAPE chemistry have been created, including those that are faster reacting and capable of interrogating RNA structure on the second timescale. During the last decade, the reactivity of multiple reagents has been extensively validated on numerous RNAs. These include, among others, 1-methyl-7-nitroisatoic anhydride (1M7) [7], 1-methyl-6-nitroisatoic anhydride (1M6) [8], 2-methyl-3-furoic acid imidazolide (FAI) and 2-methylnicotinic acid imidazolide (NAI) [9], and benzoyl cyanide (BzCN) [10], or the recently introduced 2-aminopyridine-3-carboxylic acid imidazolide (2A3), for the interrogation of RNA structures in vivo [11]. The choice of the SHAPE reagent for RNA structure probing depends on its specific features, e.g., efficiency, accuracy, or the ability to permeate biological membranes. 

In the last decade, high-throughput techniques that combine chemical probing with next-generation sequencing have started to shed new light on the sequence-structure relationship of RNA. Because of the single-nucleotide resolution measurements of the RNA structure, high-throughput sequencing-based approaches are providing genome-wide snapshots of the RNA structure. Several such techniques have been developed: SHAPE-Seq [5,6], DMS-Seq [12,13,14], MAP-Seq (Multiplexed Accessibility Probing sequencing) [15], and SHAPE-MaP (SHAPE combined with mutational profiling) [16] or DMS-Mapseq [17]. Each follows the same routine protocol, consisting of the following steps: (i) modification of the RNA depending on the structure; (ii) generating a cDNA pool via reverse transcription (RT) of the modified RNA; (iii) construction of the sequencing library; (iv) sequencing of the library; (v) bioinformatic analysis. However, they differ in the approaches for adduct detection, e.g., SHAPE-Seq uses RT conditions where an enzyme stops at the modified nucleotide, and in SHAPE-MaP reverse transcriptase incorporates noncomplementary nucleotides at the sites of SHAPE chemical adducts. The latter is possible due to the presence of Mn^2+^ during the RT reaction, as Mn^2+^ (among other divalent cations) has been shown to decrease the fidelity of the DNA synthesis [18]. This increased error frequency in the presence of Mn^2+^ has been observed in multiple reverse transcriptases (e.g., [19,20] is the main principle of the mutational profiling approaches). Moreover, several new-generation reverse transcriptases with increased robustness and thermostability allow for obtaining full-length cDNAs of highly structured RNAs, as they might incorporate the modifications into cDNA as random mutations. The most prominent example of such an enzyme is a thermostable group II intron RT (TGIRT-III), and its ability to read through the methyl adducts in DMS-MaP-Seq has been shown recently in a breakthrough publication in the *Nature Journal* [21]. 

Such sequencing-based techniques are complex since they involve many steps. Surprisingly, very little work has been done to evaluate the impact of the particular steps of the RNA chemical probing combined with sequencing on the quality of the obtained structural signal. To fill the lack of direct comparisons between current protocols, we report a simple but crucial comparison between different mRNA isolation methods, chemical reagents for structure-dependent RNA modification, and enzymes used for cDNA synthesis. We believe that the results of our analyses are likely to have a far-reaching impact on how SHAPE experiments are conducted in many laboratories.

## 2. Results

### 2.1. Evaluation of the SHAPE-MaP Library Preparation

**Figure 1:**
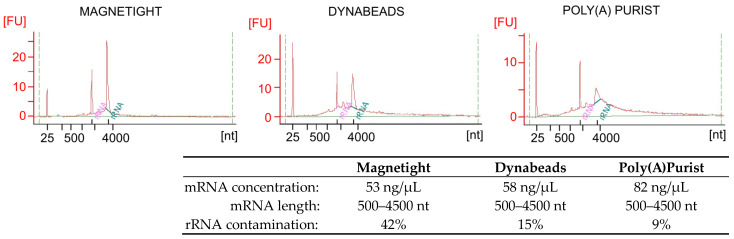
Electropherograms of the mRNA samples. RNA size [nt] and sample fluorescence [FU] are presented. Ribosomal RNA molecules are depicted in pink (18S rRNA) and green (28S rRNA). The quantitation data for the mRNA samples and contamination with ribosomal RNA are presented in the table beneath the figure.

The isolation of pure and intact mRNA is essential for sequencing technologies. We have used the RNA 6000 kit with the 2100 Bioanalyzer (Agilent Technologies, Inc., Santa Clara, CA, USA) to analyze mRNA samples purified from total HEK293 cell line RNA with three methods widely used in transcriptome analysis with sequencing technologies: Magnetight oligo (dT) particles, Dynabeads Oligo (dT)25, and Poly(A)Purist MAG kit (Figure 1).

We observed that depending on the purification process, the quality and the integrity of the mRNA preparations varied significantly. The distribution of the mRNA fragment lengths did not differ between particular preparations. The electropherograms highlight the characteristic peaks indicating contaminating ribosomal RNA (Figure 1). The mRNA purified with Magnetight oligo (dT) particles and with Dynabeads Oligo (dT)25 samples had a significant amount of contaminating ribosomal RNA. In contrast, the electropherogram for the mRNA isolated with the Poly(A)Purist MAG kit indicated that the sample was enriched in mRNA and only contained a minor amount of contaminating ribosomal RNA. Therefore, we have decided to use it for the SHAPE protocol.

**Figure 2:**
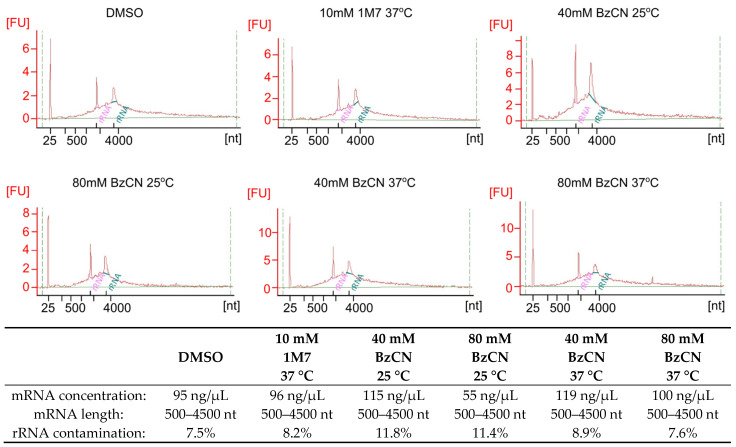
Electropherograms of mRNA samples after SHAPE modification. RNA size [nt] and sample fluorescence [FU] are presented. Ribosomal RNA molecules are depicted in pink (18S rRNA) and green (28S rRNA). The quantitation data for the mRNA samples and contamination with ribosomal RNA are presented in the table beneath the figure.

SHAPE electrophiles (1M7 or BzCN) were added to the purified and folded mRNA and incubated until the reagent had either reacted with the RNA or degraded via hydrolysis with water (five hydrolysis half-lives). A control reaction (no-reagent control) was performed in parallel. This important control measured the intrinsic background mutation rate of the reverse transcriptase under Mn^2+^ conditions, and it detected naturally occurring RNA modification events. To ensure relatively even modification of the nucleotides, we have used standard conditions of concentration and temperature for 1M7 (f.c. 10 mM at 37 °C), and four different conditions for BzCN described in the literature, with medium and high concentration (f.c. 40 mM and 80 mM, respectively) and two temperatures: 25 °C, where the half-life of BzCN is long (15 s), and 37 °C, where the half-life of BzCN is short (5 s). The reagent half-life is an essential factor in probing dynamic RNA structures since it determines the time of the structure readout. Additionally, in in vivo experiments, the cell membrane diffusion time must be considered. The quality of the modified mRNA was assessed with Agilent Bioanalyzer (Figure 2). All of the SHAPE-modified RNA samples were of good quality, and no reagent-induced or condition-dependent degradation of RNA was noticed. 

**Figure 3:**
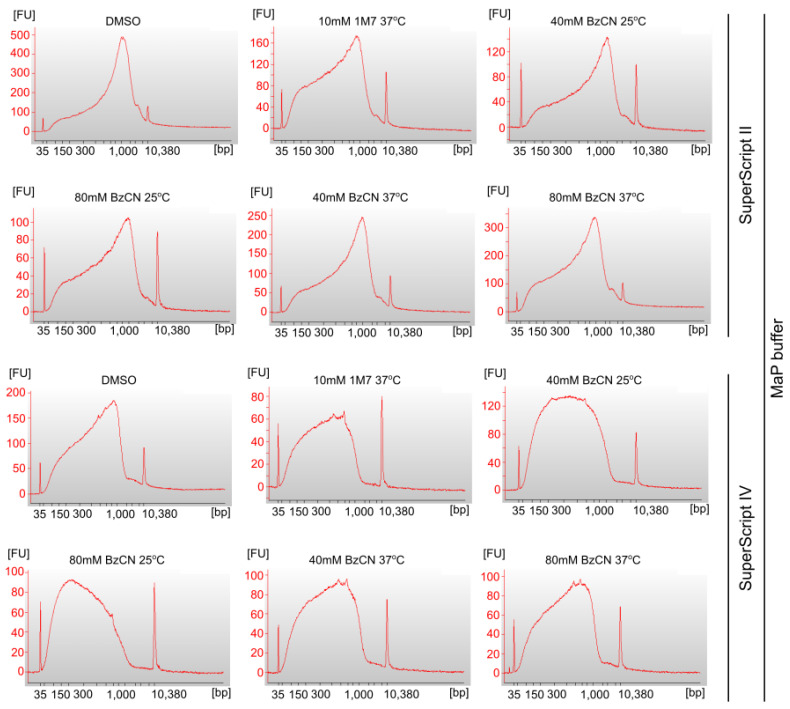
Electropherograms of dsDNA samples prepared with SuperScript II Reverse Transcriptase with a MaP buffer and SuperScript IV Reverse Transcriptase with a MaP buffer. DNA size [bp] and sample fluorescence [FU] are presented.

**Figure 4:**
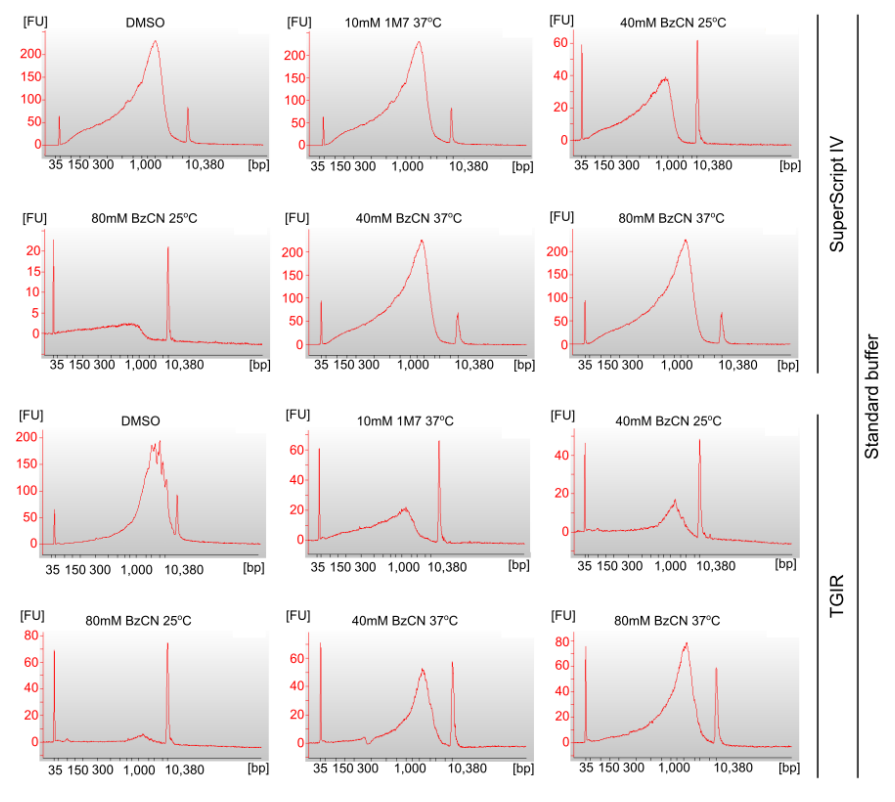
Electropherograms of dsDNA samples prepared with SuperScript IV Reverse Transcriptase with a standard buffer and TGIRT-III Reverse Transcriptase with a standard buffer. DNA size [bp] and sample fluorescence [FU] are presented.

In the attempt to optimize the signal-to-background ratio of SHAPE mutational profiling, we sought to find the best reverse transcription conditions. We performed the reverse transcription in two steps. First-strand cDNA synthesis was performed using three different enzymes, namely, SuperScript II Reverse Transcriptase (SSII), SuperScript IV Reverse Transcriptase (SSIV), or TGIRT-III Enzyme, and four different conditions as follows: SSII with Mn^2+^-containing MaP buffer (SSII-Mn^2+^), SSIV with Mn^2+^-containing MaP buffer (SSIV-Mn^2+^), SSIV with a standard SSIV buffer (SSIV), or TGIRT-III with a standard TGIRT buffer (TGIRT). These enzymes have been previously shown to generate mutational signatures in cDNA when reading through chemically modified nucleotides. Therefore, to allow for a reliable comparison of the existing SHAPE-MaP protocols, the conditions of the first-strand cDNA synthesis were based on the existing protocols for SHAPE-MaP with SSII, SSIV, and TGIRT. Second strand synthesis was performed using NEBNext Ultra II Non-Directional RNA Second Strand Synthesis Module. We assessed dsDNA’s size and concentration using an Agilent Bioanalyzer 2100 with an HS DNA kit. This analysis showed apparent differences in length distribution between dsDNAs produced with different reverse transcriptases. The dsDNA fragments produced with SSII with Mn^2+^ buffer were of average size 600–893 bp, most of which were in the length range of 500–1000 bp (Figure 3 and Table 1). SSIV with Mn^2+^ produced shorter fragments, with an average size of 390–566 bp, but with comparable amounts of products in the length range of 300–500 and 500–1000 bp. The dsDNA fragments produced with SuperScript IV Reverse Transcriptase with a standard buffer were of a size comparable to those generated with SSII with Mn^2+^ (Figure 4 and Table 1). The longest fragments were provided by the TGIRT-III Enzyme (up to 1745 bp average size), but the yield of dsDNA was the lowest.

**Figure 5:**
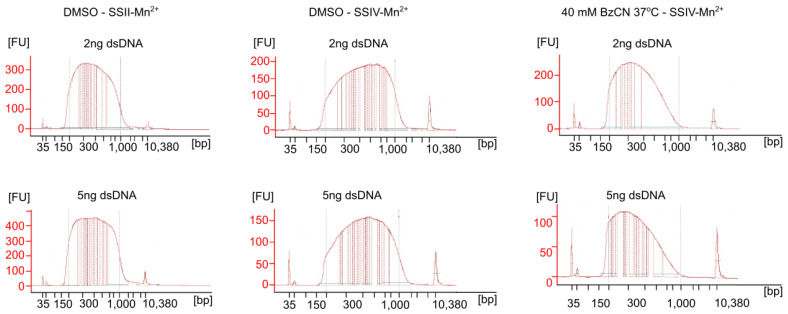
Electropherograms of the libraries prepared from 2 ng and 5 ng of dsDNA samples. DNA size [bp] and sample fluorescence [FU] are presented. Dashed lines represent a typical size distribution of the cDNA libraries (250–1000 bp).

The next step of the protocol that we analyzed was cDNA fragmentation. The enzymatic DNA fragmentation we have used is more sensitive to the DNA input than mechanical fragmentation. The Nextera XT protocol is optimized for 1 ng of input dsDNA to produce libraries of a typical size distribution of ~250–1000 bp. However, we have noticed that using 1 ng of the input dsDNA, as recommended, resulted in an overrepresentation of shorter 200–300 bp fragments. Since we decided to sequence the libraries using a paired-end setup with 150 bp read length, such fragments would result in overlapping read pairs reducing the efficiency of mutational signatures readout. The length distribution of the resulting libraries prepared from 1 ng of dsDNA is presented in Figure S1 and Table S1. Therefore, we have prepared selected libraries from higher input of dsDNA, namely, 2 ng and 5 ng. The length distribution of the resulting libraries prepared from 2 ng and 5 ng of dsDNA is presented in Figure 5. Based on these results, 5 ng of dsDNA input was optimal for library preparation due to the slightly wider fragment length plateau and the ability to provide higher library diversity and a lower PCR duplicate ratio. The length distribution of the resulting libraries prepared from 5 ng of dsDNA is presented in Figure S2 and Table S2.

### 2.2. SHAPE-MaP Sequencing Results

**Figure 6:**
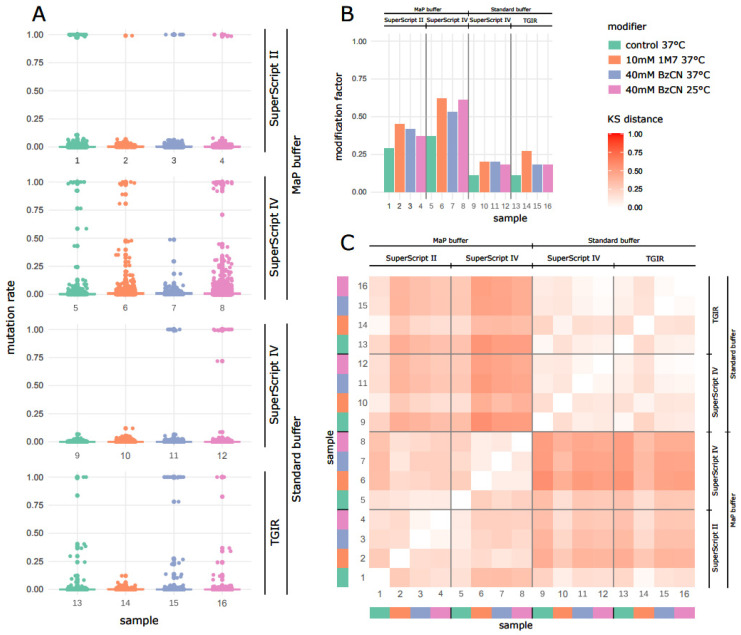
The analysis of the SHAPE-MaP sequencing results. In each graph, groups of samples were designated due to the reverse transcriptases used (SuperScript II, SuperScript IV, and TGIR) and buffers (MaP and Standard). Colors indicate the modifier (control, 1M7, and BzCN), its concentration (10 mM, 40 mM), and the treatment temperature (37 °C and 25 °C). 1, 5, 9, and 13—samples treated with DMSO; 2, 6, 10, and 14—samples treated with 10 mM 1M7 at 37 °C; 3, 7, 11, and 15—samples treated with 40 mM BzCN at 37 °C; 4, 8, 12, and 16—samples treated with 40 mM BzCN at 25 °C. (**A**) The distribution of the mutation rate values in analyzed samples. Dots represent individual nucleotides with an effective coverage ≥ 100. (**B**) Comparison of modification factors calculated as a fraction of the nucleotides with an effective coverage ≥ 100, revealing the mutation rate > 0.001. (**C**) Sample distance matrix calculated using the nucleotide mutation rates. Kolmorogov–Smirnov (KS) distance is presented in different shades of red.

The cDNA libraries prepared using the above-described conditions have been subjected to Illumina sequencing. During the analysis of the obtained sequence reads, we focused on the influence of the RT enzymes and RT conditions on the quality of the structural signal. To measure it, we have analyzed the distribution of per-base mutation frequencies (mutation rate) across the mRNA transcripts (Figure 6). The first observation was that the mRNAs reversely transcribed in Mn^2+^-containing MaP buffer presented higher mutation rates than those prepared with the standard RT buffer (Figure 6A,B). SuperScript IV with MaP buffer showed the highest mutation rates measured in both 1M7-treated samples and BzCN-treated ones, compared to the DMSO control. The DMSO control samples were affected by a mutation rate increase in MaP buffer similar to SHAPE-treated samples, which agrees with previous reports about decreased RT fidelity in the presence of Mn^2+^ ions. 

The comparison of SHAPE reagents revealed that in most of the tested RT conditions, 1M7 provided a higher structural signal than BzCN, being consistently the best, or as good as BzCN, choice. The highest gain on the mutation rate compared to control DMSO conditions were observed in the combination of 1M7 with SuperScriptIV RT enzyme and MaP buffer, suggesting the best signal-to-noise separation (median ratio ± standard deviation: 0.20 ± 0.04 for SSIV with MaP buffer, 0.12 ± 0.04 for SSII with MaP buffer, 0.11 ± 0.04 for TGIRT-III with standard buffer, and 0.08 ± 0.01 for SSIV with standard buffer). Interestingly, the distances of samples treated with 1M7, compared to standard and MaP buffers, were lower than those for BzCN (Figure 6C), suggesting lower sensitivity of SuperScript enzymes to Mn^2+^ presence during reverse transcription of 1M7 adducts.

Inspection of SHAPE-MaP signals (Figure 6A–C) confirmed the higher ability of SSIV with Mn^2+^-containing MaP buffer to translate 1M7 SHAPE adducts on structurally flexible bases into the mutational signatures. Altogether, these results identified SSIV in Mn^2+^-containing buffer as the optimal RT enzyme for SHAPE-MaP experiments.

## 3. Discussion

After its publication in 2014 [16], the SHAPE-MaP protocol became the most popular tool for experimental studies on RNA secondary structure. In this study, we decided to validate in detail the influence of the SHAPE reagent, buffer conditions, and the type of reverse transcriptase on the efficiency of the protocol. During the development of the original SHAPE-MaP protocol, manganese divalent cations were found to most efficiently promote SSII readthrough and mutational detection of 1M7, 1M6, and NMIA chemical adducts [16]. The results of our study confirmed that the employment of Mn^2+^-containing MaP buffer is a major factor providing a significant increase in the structural signal.

We have observed that all reverse transcriptases suitable for SHAPE-Map protocol (SSII, SSIV, and TGIRT-III) could introduce mutations at the positions modified by both tested reagents (1M7 and BzCN); however, SSIV with a MaP buffer achieved the highest signal-to-noise ratio. One of the explanations could be the higher processivity and reaction speed of the SuperScript IV RT over its predecessor, SSII. Our observation is also consistent with the general high activity of the Moloney Murine Leukemia Virus Reverse Transcriptase (M-MuLV RT) in the presence of Mn^2+^ and the ability of the manganese cations to advocate a mutagenic nature of the DNA polymerases [22], since SS RT enzymes are M-MuLV RT mutants.

However, it was surprising to observe that the length of obtained cDNA was negatively affected by the RT in a buffer containing Mn^2+^ (Figure 3 and Figure 4). Recent research on mutational detection of natural RNA modifications evaluated the performance of different reverse transcriptases on detecting *Saccharomyces cerevisiae* tRNA modifications in the presence of manganese cations [18]. The authors have used SuperScript III, SuperScript IV, ProtoScript II, and EpiScript RT. They generally observed that Mn2+ increased the modification-induced mutation rates and nucleotide skipping, accompanied by increased readthrough as represented by longer cDNA products. This difference may be related to the different nature of detected modifications—SHAPE regents introduce adducts on a 2′-OH group of the ribose, whereas tRNA modifications analyzed by Kristen et al. contained mostly nucleobase adducts. Furthermore, we also observed the differences in cDNA length between 1M7 and BzCN adduct-containing products. Therefore, the role of Mn^2+^ in the modulation of the mutational adduct detection and readthrough depends on the localization and the type of the adduct.

The 1M7 was initially identified as a general purpose SHAPE reagent because of its short reaction half-life of 14 s and its ability to address the flexibility of all four ribonucleotides with a similar reactivity [23]. BzCN, on the other hand, is supremely suited for time-resolving RNA structures due to its extremely short but still manageable half-life of 0.25 s at 37 °C [10]. To our knowledge, a direct comparison of the reactivity of these two SHAPE reagents has never been tested before for RNA pools. Only one study tackled this point, testing the reactivity of 1M7 and BzCN on one isolated RNA molecule, RNase P RNA [10]. That was the first experiment reporting BzCN utility in SHAPE, by Mortimer and Weeks, and they reported a robust correlation between 1M7 and BzCN reactivity on RNase P RNA. We have, therefore, examined the adduct detection rates of 1M7 and BzCN-treated human mRNAs. It appeared that BzCN showed a pattern of MaP mutation rates markedly different from that of 1M7. In general, 1M7 generated more robust mutation profiles, and it produced more structured information than BzCN. Overall, these results demonstrate that 1M7 is a robust human mRNA modifier in vitro, whereas BzCN performs less efficiently. These differences between the reagents observed in our study might be due to the type of RNA we used rather than the type of mRNA modification protocol since BzCN has been previously shown to be a potent in vitro modification reagent [10,24].

Altogether, our results reveal that the latest member of the SuperSscript RT family, SSIV, in combination with Mn^2+^-containing buffer, outperforms SSII in the mutational profiling of human mRNAs. However, the high sensitivity of mutational detection is coupled with an Mn^2+^-induced decrease of a readthrough of SHAPE adducts, revealing a novel role of Mn^2+^ in the modulation of reverse transcriptase activity.

## 4. Materials and Methods

### 4.1. Cell Line

HEK293 cells were obtained from ATCC and maintained in DMEM (Biowest, Nuaillé, France) supplemented with 10% FBS, 10,000 units/mL penicillin G, 10 mg/mL streptomycin sulfate, and 25 μg/mL amphotericin B.

### 4.2. RNA Isolation

Total RNA was isolated using TRI Reagent (Mercator Medical S.A., Kraków, Poland), following the manufacturer’s instruction, and stored at −80 °C. The concentration of the total RNA was measured with a Qbit fluorometer using RNA HS assay. The quality of the total cellular RNA was verified using an Agilent Bioanalyzer 2100 with an RNA Nano 6000 kit (Total RNA assay, Agilent Technologies, Inc., Santa Clara, CA, USA). The mRNA was isolated from the total RNA with Magnetight oligo (dT) particles (Merck, Darmstadt, Germany), Dynabeads Oligo (dT)25 (Thermo Fisher Scientific Inc., Waltham, MA, USA), or Poly(A)Purist MAG kit (Thermo Fisher Scientific Inc., Waltham, MA, USA), following the manufacturer’s instruction, and stored at −80 °C. The concentration, integrity, and purity of the mRNA samples were assessed using an Agilent Bioanalyzer 2100 with RNA Nano 6000 kit (mRNA assay, Agilent Technologies, Inc., Santa Clara, CA, USA).

### 4.3. Chemical Modification of RNA

A total of 1 µg of purified mRNA was folded by a 95 °C denaturation, a quick cool to 4 °C, followed by 20 min incubation at 37 °C in 100 mM HEPES pH 8.0, 10 mM MgCl_2,_ and 100 mM NaCl. Samples were then modified for 5 hydrolysis half-lives of the SHAPE reagents: 5 min at 37 °C with 10 mM 1-methyl-7-nitroisatoic anhydride (1M7), 15 s at 25 °C with 40 mM or 80 mM benzoyl cyanide (BzCN), and 5 s at 37 °C with 40 mM or 80 mM BzCN. A control sample containing DMSO instead of a SHAPE reagent was included. Modified RNA was then cleaned up and eluted in RNase-free water using RNA Clean and Concentrator-5 (Zymo Research, Irvine, CA, USA) and stored at −20 °C. The concentration, integrity, and purity of modified mRNA samples were assessed using an Agilent Bioanalyzer 2100 with an RNA Nano 6000 kit (mRNA assay, Agilent Technologies, Inc., Santa Clara, CA, USA).

### 4.4. Reverse Transcription

A total of 200 ng of the modified RNA was subjected to reverse transcription with random 9-mer primers (New England Biolabs, Ipswich, MA, USA). Primers were annealed by incubation at 65 °C for 5 min. We have used the following protocols: SuperScript II Reverse Transcriptase (SSII, Thermo Fisher Scientific) with Mn^2+^ buffer (SSII-Mn^2+^), SuperScript IV Reverse Transcriptase (SSIV, Thermo Fisher Scientific Inc., Waltham, MA, USA) with Mn^2+^ buffer (SSIV-Mn^2+^), and a standard SSIV buffer (SSIV) and TGIRT-III Enzyme (InGex, St Louis, MO, USA) with a standard TGIRT buffer (TGIRT, InGex, St Louis, MO, USA)). The conditions of reverse transcription were as follows.

**SSII-Mn^2+^:** A total of 8 μL freshly prepared Mn^2+^-containing MaP buffer (50 mM Tris pH 8.0, 75 mM KCl, 6 mM MnCl_2_, 10 mM DTT, and 0.5 mM dNTPs) was added to the RNA/primer mixture and incubated at 25 °C for 2 min, followed by the addition of 200 U Superscript II. The reaction mixtures (total volume of 20 μL) were incubated at 25 °C for 10 min, 42 °C for 3 h, and 70 °C for 15 min. Samples were held on ice.

**SSIV-Mn^2+^:** A total of 8 μL freshly prepared Mn^2+^-containing MaP buffer (50 mM Tris pH 8.0, 75 mM KCl, 6 mM MnCl_2_, 10 mM DTT, and 0.5 mM dNTPs) was added to the RNA/primer mixture and incubated at 25 °C for 2 min, followed by the addition of 200 U Superscript IV. The reaction mixtures (total volume of 20 μL) were incubated at 25 °C for 10 min, 42 °C for 3 h, and 70 °C for 15 min. Samples were held on ice.

**SSIV:** A total of 4 μL buffer (50 mM Tris-HCl pH 8.3, 75 mM KCl, and 3 mM MgCl_2_), 1 μL 100 mM DTT, 1 μL 10 mM dNTP, and 200 U Superscript IV was added to the RNA/primer mixture. The complete reaction mixtures (total volume of 20 μL) were incubated at 25 °C for 10 min, 55 °C for 10 min, and 70 °C for 15 min. Samples were held on ice.

**TGIRT:** A total of 8 μL buffer (50 mM Tris-HCl pH 8.3, 75 mM KCl, and 3 mM MgCl_2_) was added to the RNA/primer mixture and incubated at 25 °C for 5 min, followed by the addition of 1 μL 100 mM DTT, 2 μL 10 mM dNTP mix and 200 U TGIRT-III Enzyme. The reaction mixtures (total volume of 20 μL) were incubated at 57 °C for 1.5 h and 70 °C for 15 min. Samples were held on ice.

### 4.5. Second-Strand Synthesis

Reverse transcription reactions were purified using Microspin™ G-50 Columns (GE Healthcare, Chicago, IL, USA). Second strand synthesis was performed using NEBNext Ultra II Non-Directional RNA Second Strand Synthesis Module (New England Biolabs, Ipswich, MA, USA) with incubation at 16 °C for 2.5 h. The dsDNA from the second-strand synthesis reaction was purified using PureLink PCR micro spin columns (Thermo Fisher Scientific Scientific Inc., Waltham, MA, USA), according to the manufacturer’s instructions. The size and concentration of dsDNA were assessed using an Agilent Bioanalyzer 2100 with an HS DNA kit (Agilent Technologies, Inc., Santa Clara, CA, USA).

### 4.6. Library Preparation and Sequencing

Approximately 1–5 ng of purified dsDNA was fragmented and tagged with adapter sequences in a single step using Nextera XT DNA Library Preparation Kit (Illumina Inc., San Diego, CA, USA) at 55 °C for 5 min, followed by incubation at 10 °C and sample neutralization, according to the manufacturer’s instruction. Tagmented libraries were then used as inputs in PCR reactions using 14 cycles of PCR amplification. PCR adds the Index 1 (i7), Index 2 (i5), and full adapter sequences to the tagmented DNA. The index adapters and Nextera PCR Master Mix were added directly to the 25 μL of tagmented dsDNA from the previous step. PCR reactions were cleaned up with Agencourt Ampure XP (Beckman Coulter, Brea, CA, USA) beads following the manufacturer’s protocol, eluting with 15 μL RNAse-free water. No direct size selection was performed on the resulting adapter-ligated library. Libraries were assayed for quality using an Agilent Bioanalyzer 2100 HS DNA chip. Libraries were then sequenced on an Illumina MiSeq platform following the manufacturer’s standard cluster generation and sequencing protocols.

### 4.7. SHAPE-Seq Data Analysis

Fastq files generated from the Illumina sequencing were mapped to the human transcriptome GRCh38.p12 using bowtie2 [25]. The average transcript coverage was analyzed with Samtools [26]. Transcripts with an average coverage of ≥50.0 were further considered. The mutation rates were calculated using the ShapeMapper 2.1.3 software (https://weekslab.com/software/) [8]. As the ShapeMapper operates simultaneously on the control and the treated sample, for each pair of control and treated samples, only transcripts that met the coverage criteria in both samples were processed. The statistics of the mutation rate were limited to the bases with an effective coverage ≥100. The modification factors were calculated as the fraction of the nucleotides with an effective coverage ≥100, revealing the mutation rate as >0.001. A matrix of sample distances based on the mutation rate distributions was calculated as previously published [27].

## Figures and Tables

**Table 1:** The quantitation data for dsDNA samples. To allow for a reliable comparison of the existing SHAPE-MaP protocols, the conditions of the reverse transcription (MaP buffer or Standard buffer) were based on the existing protocols for SHAPE-MaP with SSII, SSIV, or TGIR.

	DMSO	10 mM 1M7 37 °C	40 mM BzCN 25 °C	80 mM BzCN 25 °C	40 mM BzCN 37 °C	80 mM BzCN 37 °C		
Concentration [ng/µL]	17.2	9.0	5.7	5.0	9.6	16.0	SSII	MaP buffer
Average size [bp]	893	600	687	615	701	670		
Concentration [ng/µL]	14.7	4.9	10.8	6.2	7.9	7.2	SSIV	
Average size [bp]	566	425	390	344	438	479		
Concentration [ng/µL]	8.4	3.6	1.4	0.5	14.1	7.8	SSIV	Standard buffer
Average size [bp]	725	394	644	549	718	507		
Concentration [ng/µL]	4.2	1.1	0.3	0.2	1.9	1.6	TGIR	
Average size [bp]	1745	642	644	549	1131	984		

## Data Availability

The data for this study have been deposited in the European Nucleotide Archive (ENA) at EMBL-EBI under the accession number PRJEB60419.

## Supplementary Materials

The following supporting information can be downloaded at: https://www.mdpi.com/article/10.3390/ijms24097890/s1.

## References

[1] Schroeder R., Grossberger R., Pichler A., Waldsich C. (2002). RNA folding in vivo. Curr. Opin. Struct. Biol..

[2] Cannons A.C., Cannon J. (2001). The stability of the Chlorella nitrate reductase mRNA is determined by the secondary structure of the 5′-UTR: Implications for posttranscriptional regulation of nitrate reductase. Planta.

[3] Song L., Axtell M.J., Fedoroff N.V. (2010). RNA Secondary Structural Determinants of miRNA Precursor Processing in Arabidopsis. Curr. Biol..

[4] Weeks K.M. (2010). Advances in RNA structure analysis by chemical probing. Curr. Opin. Struct. Biol..

[5] Lucks J.B., Mortimer S.A., Trapnell C., Luo S., Aviran S., Schroth G.P., Pachter L., Doudna J.A., Arkin A.P. (2011). Multiplexed RNA structure characterization with selective 2′-hydroxyl acylation analyzed by primer extension sequencing (SHAPE-Seq). Proc. Natl. Acad. Sci. USA.

[6] Mortimer S.A., Trapnell C., Aviran S., Pachter L., Lucks J.B. (2012). SHAPE-Seq: High-throughput RNA structure analysis. Curr. Protoc. Chem. Biol..

[7] Rice G.M., Leonard C.W., Weeks K.M. (2014). RNA secondary structure modeling at consistent high accuracy using differential SHAPE. RNA.

[8] Busan S., Weeks K.M. (2017). Accurate detection of chemical modifications in RNA by mutational profiling (MaP) with ShapeMapper 2. RNA.

[9] Spitale R.C., Crisalli P., Flynn R.A., Torre E.A., Kool E.T., Chang H.Y. (2013). SHAPE analysis in living cells. Nat. Chem. Biol..

[10] Mortimer S.A., Weeks K.M. (2008). Time-Resolved RNA SHAPE Chemistry. J. Am. Chem. Soc..

[11] Marinus T., Fessler A.B., Ogle C.A., Incarnato D. (2021). A novel SHAPE reagent enables the analysis of RNA structure in living cells with unprecedented accuracy. Nucleic Acids Res..

[12] Ding Y., Tang Y., Kwok C.K., Zhang Y., Bevilacqua P.C., Assmann S.M. (2013). In vivo genome-wide profiling of RNA secondary structure reveals novel regulatory features. Nature.

[13] Rouskin S., Zubradt M., Washietl S., Kellis M., Weissman J.S. (2014). Genome-wide probing of RNA structure reveals active un-folding of mRNA structures in vivo. Nature.

[14] Talkish J., May G., Lin Y., Woolford J.L., McManus C.J. (2014). Mod-seq: High-throughput sequencing for chemical probing of RNA structure. RNA.

[15] Seetin M.G., Kladwang W., Bida J.P., Das R. (2014). Massively parallel RNA chemical mapping with a reduced bias MAP-seq pro-tocol. Methods Mol. Biol..

[16] Siegfried N.A., Busan S., Rice G.M., Nelson J.A., Weeks K.M. (2014). RNA motif discovery by SHAPE and mutational profiling (SHAPE-MaP). Nat. Methods.

[17] Zubradt M., Gupta P., Persad S., Lambowitz A.M., Weissman J.S., Rouskin S. (2017). DMS-MaPseq for genome-wide or targeted RNA structure probing in vivo. Nat. Methods.

[18] Kristen M., Plehn J., Marchand V., Friedland K., Motorin Y., Helm M., Werner S. (2020). Manganese Ions Individually Alter the Reverse Transcription Signature of Modified Ribonucleosides. Genes.

[19] Sirover M.A., Loeb L.A. (1977). On the fidelity of DNA replication. Effect of metal activators during synthesis with avian myelo-blastosis virus DNA polymerase. J. Biol. Chem..

[20] Cases-Gonzalez C.E., Gutierrez-Rivas M., Ménendez-Arias L. (2000). Coupling ribose selection to fidelity of DNA synthesis. The role of Tyr-115 of human immunodeficiency virus type 1 reverse transcriptase. J. Biol. Chem..

[21] Tomezsko P.J., Corbin V.D.A., Gupta P., Swaminathan H., Glasgow M., Persad S., Edwards M.D., Mcintosh L., Papenfuss A.T., Emery A. (2020). Determination of RNA structural diversity and its role in HIV-1 RNA splicing. Nature.

[22] Beckman R.A., Mildvan A.S., Loeb L.A. (1985). On the fidelity of DNA replication: Manganese mutagenesis in vitro. Biochemistry.

[23] Mortimer S.A., Weeks K.M. (2007). A Fast-Acting Reagent for Accurate Analysis of RNA Secondary and Tertiary Structure by SHAPE Chemistry. J. Am. Chem. Soc..

[24] Mortimer S.A., Weeks K.M. (2009). Time-resolved RNA SHAPE chemistry: Quantitative RNA structure analysis in one-second snapshots and at single-nucleotide resolution. Nat. Protoc..

[25] Langmead B., Salzberg S.L. (2012). Fast gapped-read alignment with Bowtie 2. Nat. Methods.

[26] Danecek P., Bonfield J.K., Liddle J., Marshall J., Ohan V., Pollard M.O., Whitwham A., Keane T., McCarthy S.A., Davies R.M. (2021). Twelve years of SAMtools and BCFtools. GigaScience.

[27] Dodge Y. (2008). Kolmogorov–Smirnov Test. The Concise Encyclopedia of Statistics.

