# Supplementary material for: The Evaluation of SHAPE-MaP RNA Structure Probing Protocols Reveals a Novel Role of Mn2+ in the Detection of 2′-OH Adducts
Source: Int J Mol Sci. 2023 Apr 26;24(9):7890. doi: 10.3390/ijms24097890 (PMC10178110; doi:10.3390/ijms24097890)
Supplement: Supplementary file 1 [file ijms-24-07890-s001.zip › ijms-2294444-supplementary.pdf]

SUPLEMENTARY FIGURES

Systematic evaluation of SHAPE-MaP RNA structure probing protocol reveals a novel role of Mn^{2+} in the detection of 2′-OH adducts

Kamilla Grzywacz [1,*], Agnieszka Chełkowska-Pauszek [2], Marianna Plucinska-Jankowska [2] and Marek Żywicki [2,*]

[1] Institute of Bioorganic Chemistry Polish Academy of Sciences, Poznań, Poland; kamilla.grzywacz@ibch.poznan.pl
[2] Department of Computational Biology, Institute of Molecular Biology and Biotechnology, Adam Mickiewicz University, Poznań, Poland; agnieszka.chelkowska@amu.edu.pl (A.C.-P.); marianna.plucinska@amu.edu.pl (M.P.-J.); marek.zywicki@amu.edu.pl (M.Ż.) * Correspondence: kamilla.grzywacz@ibch.poznan.pl (K.G.); marek.zywicki@amu.edu.pl (M.Ż.)

Figure S1. Electropherograms of the libraries prepared from 1 ng of dsDNA samples. DNA size [bp] and sample fluorescence [FU] is presented. 1, 7, 13, 19 – samples treated with DMSO; 2, 8, 14, 20 – samples treated with 10 mM 1M7 at 37°C; 3, 9, 15, 21 - samples treated with 40 mM BzCN at 25°C; 4, 10, 16, 22 - samples treated with 80 mM BzCN at 25°C; 5, 11, 17, 23 - samples treated with 40 mM BzCN at 37°C; 6, 12, 18, 24 - samples treated with 80 mM BzCN at 37°C.

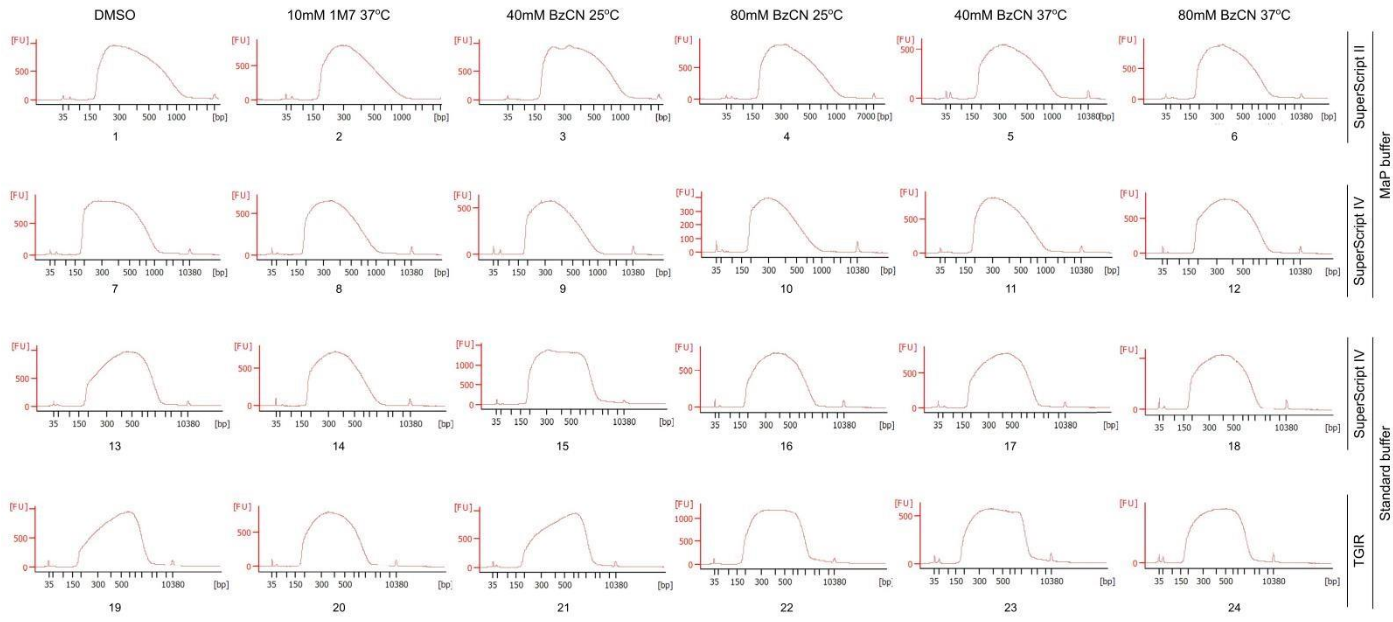

Figure S2. Electropherograms of the libraries prepared from 5 ng of dsDNA samples. DNA size [bp] and sample fluorescence [FU] is presented. 1, 7, 13, 19 – samples treated with DMSO; 2, 8, 14, 20 – samples treated with 10 mM 1M7 at 37°C; 3, 9, 15, 21 - samples treated with 40 mM BzCN at 25°C; 4, 10, 16, 22 - samples treated with 80 mM BzCN at 25°C; 5, 11, 17, 23 - samples treated with 40 mM BzCN at 37°C; 6, 12, 18, 24 - samples treated with 80 mM BzCN at 37°C.

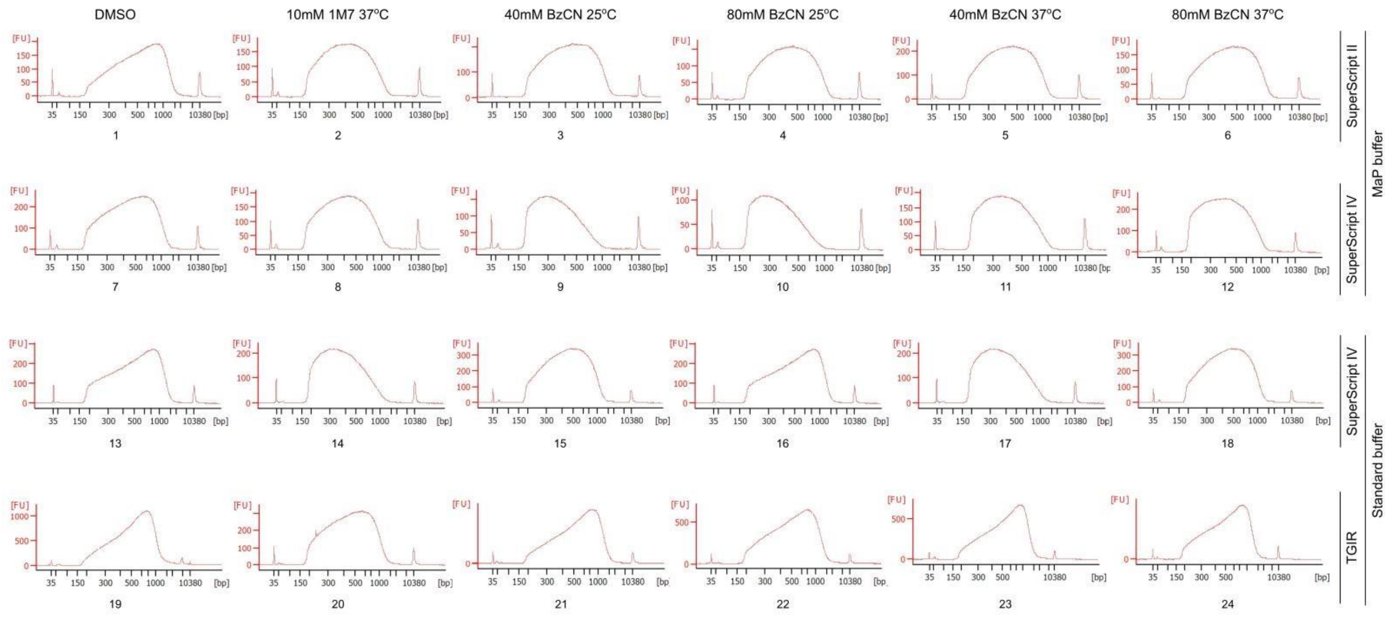

Table S1. The quantitation data for cDNA libraries prepared with 1 ng of dsDNA.

		10mM	40mM	80mM	40mM	80mM		
DMSO 1M7	BzCN	BzCN	BzCN BzCN	37°C	25°C 25°C	37°C 37°C		
Concentration [ng/µl]	56.6	37.8	53.0	44.0	26.8	44.2	SSII	MaP buffer
Average size [bp]	403	374	405	391	409	405		
Concentration [ng/µl]	48.4	29.0	29.0	13.6	41.6	24.4	SSIV	
Average size [bp]	406	388	383	364	387	403		
Concentration [ng/µl]	36.3	23.6	44.8	30.6	37.0	20.8	SSIV	Standard buffer
Average size [bp]	448	389	433	416	430	422		
Concentration [ng/µl]	31.6	31.0	37.8	43.8	36.6	20.0	TGIR	
Average size [bp]	476	417	481	442	458	451		

Table S2. The quantitation data for cDNA libraries prepared with 5 ng of dsDNA.

		10mM	40mM	80mM	40mM	80mM	DMSO 1M7	
		BzCN	BzCN BzCN	BzCN	37°C	25°C 25°C	37°C 37°C	
Concentration [ng/µl]	7.1	8.4	10.3	8.1	9.3	8.6	SSII	MaP buffer
Average size [bp]	510	433	459	449	453	461		
Concentration [ng/µl]	9.6	7.2	6.6	4.8	2.2	12.6	SSIV	
Average size [bp]	476	432	371	362	401	421		
Concentration [ng/µl]	11.0	10.1	15.8	19.0	25.2	12.7	SSIV	Standard buffer
Average size [bp]	506	401	466	444	471	473		
Concentration [ng/µl]	29.8	11.7	14.3	18.3	23.6	16.5	TGIR	
Average size [bp]	526	473	533	511	430	517		
